# Resveratrol Inhibits High Glucose-Induced H9c2 Cardiomyocyte Hypertrophy and Damage via RAGE-Dependent Inhibition of the NF-*κ*B and TGF-*β*1/Smad3 Pathways

**DOI:** 10.1155/2022/7781910

**Published:** 2022-02-25

**Authors:** Yanzhou Zhu, Fuling Wu, Qin Yang, Haixing Feng, Dingli Xu

**Affiliations:** ^1^State Key Laboratory of Organ Failure Research, Department of Cardiology, Nanfang Hospital, Southern Medical University, Guangzhou 510515, China; ^2^Department of Pharmacy, Nanfang Hospital, Southern Medical University, Guangzhou 510515, China

## Abstract

Hyperglycaemia is associated with the development of cardiac vascular disease. Resveratrol (RES) is a naturally occurring polyphenolic compound that possesses many biological properties, including anti-inflammatory properties and antioxidation functions. Our study aimed to explore the RES's protective roles on high glucose (HG)-induced H9c2 cells and the underlying mechanisms. Small-molecule inhibitors, western blotting (WB), as well as reverse-transcription PCR (RT-PCR) were employed to investigate the mechanisms underlying HG-induced damage in H9c2 cells. RES (40 *μ*g/mL) treatment significantly alleviated HG-induced cardiac hypertrophy and cardiac dysfunction. RES abated the HG‐induced increase in the levels of extracellular matrix (ECM) components and inflammatory cytokines, reducing ECM accumulation and inflammatory responses. Additionally, RES administration prevented HG‐induced mitochondrion‐mediated cardiac apoptosis of myocardial cells. In terms of mechanisms, we demonstrated that RES ameliorated the HG‐induced overexpression of receptor for advanced glycation endproducts (RAGE) and downregulation of NF-*κ*B signalling. Moreover, RES inhibited HG‐induced cardiac fibrosis by inhibiting transforming growth factor beta 1 (TGF‐*β*1)/Smad3‐mediated ECM synthesis in cultured H9c2 cardiomyocytes. Further studies revealed that the effects of RES against HG‐induced upregulation of NF-*κ*B and TGF‐*β*1/Smad3 pathways were similar to those of FPS-ZM1, a RAGE inhibitor. Collectively, the results implied that RES might help alleviate HG‐induced cardiotoxicity via RAGE‐dependent downregulation of the NF-*κ*B and TGF‐*β*/Smad3 pathways. This study provided evidence that RES can be developed as a promising cardioprotective drug.

## 1. Introduction

The global incidence of diabetes is increasing annually. Diabetic complications can affect the great majority of the body's important organs, the cardiovascular system in particular, which is the most vulnerable and results in the most serious complications. Cardiac failure causes the most deaths in diabetic patients with diabetes. One of the characteristics of diabetes is elevated blood glucose levels, a key risk factor for inducing cardiac vascular disease. It is reported that cardiovascular injury obviously contributes to increasing mortality in diabetic patients [1]. Hyperglycaemia plays an important role in cardiac hypertrophy, inflammation, fibrosis, and apoptosis, thereby resulting in heart failure by activating various signals. Hyperglycaemia majorly contributes to the development of diabetic cardiomyopathy, which triggers a set of related signalling pathways that result in cardiomyocyte apoptosis, superoxide injury, and cardiac dysfunction [2]. Accumulating studies have proved that hyperglycaemia is closely linked with a significant increase in oxidative and inflammatory factors in cardiovascular tissues through activating the nuclear factor-*κ*B (NF-*κ*B) and mitogen-activated protein kinase (MAPK) pathways [3, 4]. In addition, HG level activated the mitochondrial apoptotic pathway and induced cardiomyocyte death, which was accompanied with the expression changes of various apoptotic proteins, including Bax, Bcl-2, and caspase-3 [5]. Therefore, drugs with multiple activities, including anti-inflammatory, antioxidant, and antiapoptosis, may protect cardiomyocytes from HG-induced damage.

The receptor for advanced glycation endproducts (RAGE) was first regarded as the receptor for signal transducing of advanced glycation endproducts (AGEs) [6]. Due to persistent oxidative stress and hyperglycaemia, increased AGE formation accompanied with the hyperactivity of RAGE has been shown to be a key pathway for microvascular and macrovascular complications in diabetes [7]. High blood sugar can result in the formation of AGEs, which activates RAGE, induces an inflammatory response in the heart, and promotes the development of diabetic cardiomyopathy [8]. Currently, there exist many theories about the mechanism underlying the complications of the cardiovascular system of diabetes. Among them, AGEs and their receptor RAGE produced in a high-glucose environment have become research hotspots in recent years. The roles of AGEs, RAGE, and reactive oxygen species in developing atherosclerosis have been confirmed by many experiments; however, their roles and underlying pathways in diabetic cardiomyopathy remain unclear. Furthermore, immoderate accumulation and sedimentation of extracellular matrix (ECM) is the principal pathological change in diabetic cardiomyopathy, leading to matrix dilation, thickening of the basal membrane of the heart, and cardiomyocyte fibrosis [9]. Therefore, inhibition of inflammation and ECM accumulation are crucial targets for treating diabetic cardiomyopathy.

Resveratrol, a nonflavonoid polyphenolic compound, is widely distributed in different plants, including medicinal and edible plants, such as *Polygonum cuspidatum*, *Cassia*, grapes, and peanuts. Apart from suppressing platelet activation and refraining or slowing the development of various diseases, including cancer and cardiovascular disease, resveratrol also has several benefits, such as antioxidant, anti-inflammatory, and antiapoptotic activities [10, 11]. However, its effects on cardiac hypertrophy are yet to be ascertained.

This study investigated the influences of RES on cardiac hypertrophy and cardiac dysfunction along with the RAGE-mediated expression and secretion of inflammatory factors and ECM components in HG-treated H9c2 cells and the corresponding potential mechanisms.

## 2. Materials and Methods

### 2.1. Reagents

H9c2 cells were obtained from the Cell Bank of the Chinese Academy of Sciences (Shanghai, China). Enzyme-linked immunosorbent assay (ELISA) kits (IL-6 and TNF-*α*) were obtained from Wuhan Huamei Biological Engineering Co., Ltd. (Wuhan, Hubei, China). Rabbit polyclonal antibodies specific for ANP, *β* -MHC, NF-*κ*B p-p65, p65, and GAPDH were obtained from Cell Signaling Technology (Danvers, MA, USA). Rabbit polyclonal antibodies to RAGE, alpha smooth muscle actin (a-SMA), Bax, Bcl-2, cleaved caspase 3, TGF-*β*1, p-Smad3, and Smad3 antibodies were obtained from Abcam Biotechnology (Cambridge, UK). FPS-ZM1 was obtained from MedChem Express Co., Ltd. (New Jersey, USA). TRIzol reagent was obtained from Invitrogen (Carlsbad, CA, USA), and PrimeScript™RT Master Mix and SYBR Premix Ex Taq were obtained from TaKaRa (Dalian, China). The Annexin V-EGFP Apoptosis Detection Kit was obtained from KeyGen BioTECH (Nanjing, China). Dulbecco's modified Eagle's medium (DMEM), fetal bovine serum (FBS), and antibiotics (100 mg/mL streptomycin and 100 U/mL penicillin) were obtained from GIBCO (Grand Island, NY, USA).

### 2.2. Cell Culture

We cultured H9c2 cells in DMEM (Gibco) containing 10% foetal bovine serum (FBS) (Gibco) and 1% antibiotics (100 U/ml penicillin and 100 *μ*g/mL streptomycin from Beyotime, Jiangsu, China) (under humidified conditions of 5% CO_2_ at 37°C). High glucose was used to treat cells (HG, 25 mM D-glucose) for 24 h to set up the cell toxic model. A CCK-8 kit was used to study the role of RES on the viability of cells. We seeded H9c2 cells into plates containing 96 wells (2 × 105 cells per well). When 24 h of incubation was completed, treatment with various RES concentrations (0–400 *μ*g/mL) was performed on cells for 24 h in triplicate. To explore the effect of RES *in vitro*, H9c2 cells were treated beforehand with 20 and 40 *μ*g/mL RES or 100 *μ*M FPS-ZM1 (MedChem Express).

### 2.3. Determination of Total Protein Content in H9c2 Cell

H9c2 cells were subjected to treatment with solutions of various HG concentrations. Next, the cells were rinsed using PBS, trypsinised, and counted with a haemocytometer. We lysed an equal amount of cells in RIPA buffer which contained 0.1% (w/v) SDS, 0.5% (w/v) sodium deoxycholate, and 1.0% (w/v) Nonidet P-40 (in PBS). After allowing the cells to rest for 15 min at 4°C, the cells were subjected to centrifugation (12 000 revolutions per minute at four degrees Celsius for 10 min) for collecting the supernatant. The supernatant's total protein content was quantified using the BCA method.

### 2.4. Western Blot Analysis

We seeded H9c2 cells (2 × 10^6^ cells per well) into plates of six wells and incubated them for 24 h. Then, the cells were subjected to treatment with different RES and HG concentrations and underwent incubation for 24 h. Western blotting (WB) was performed as stated previously [12]. Total protein extraction from the treated cells was conducted using a kit (KeyGen Biotech, Nanjing, China). In brief, the cells were lysed in RIPA lysis buffer containing inhibitors of phosphatase and protease and subjected to centrifugation (12 000 revolutions per minute at four degrees Celsius for 10 min) for collecting the protein. 8% SDS-PAGE was employed to separate an equal amount of protein, which was subsequently transported to activated membranes of PVDF. Next, we blocked these membranes at room temperature with 5% BSA for 1 hour and hybridized them with the corresponding primary antibodies specific for ANP (1 : 1000), *β* -MHC (1 : 2000), Bcl‐2 (1 : 500), Bax (1 : 1000), RAGE (1 : 200), cleaved caspase‐3 (1 : 1000), NF-*κ*B (1 : 1000), TGF‐ *β* 1 (1 : 1000), Smad3 (1 : 1000), p‐Smad3 (1 : 1000), *α* -SMA (1 : 1000), collagen 1 (1 : 1000), and GAPDH (1 : 2000) at overnight at 4°C. Subsequently, the membranes were rinsed using TBST  and then subjected to incubation with the corresponding secondary antibody (1 : 5000). The protein brands were visualised with an electrochemical luminescence (ECL) kit in the system for automatic chemiluminescence image analysis (Tanon, Shanghai, China). Protein levels were normalised in comparison to those of the loading GADPH control. Experiments were repeated three times.

### 2.5. Cell Apoptosis Assay

We stained H9c2 cells with the Annexin V‐FITC Apoptosis Kit (KeyGEN, Jiangsu, China) following the protocols of manufacturer. The treated cells were rinsed using PBS, trypsinised with trypsin without EDTA, and subjected to centrifugation at 2000 revolutions per minute for 5 min at room temperature to harvest cells. We stained the obtained cells with 5 *μ*L Annexin V-FITC and 5 *μ*L of PI in a diluted solution and incubated them at room temperature in the dark for a quarter. Later, the stained cells were further detected and analysed using flow cytometry within half an hour (FACS Calibur, Bio-Rad Laboratories, Inc., USA). The lower left, upper left, lower right, and upper right quadrants, respectively, represent healthy, dead, early apoptotic, and late apoptotic cells.

### 2.6. Real-Time Reverse-Transcription PCR [12]

After treatment, we extracted total RNA from H9c2 cells utilizing TRIzol reagent (Invitrogen, Carlsbad, CA, USA) in accordance with the instructions of manufacture. RNA was reverse‐transcribed using oligo (dT) primers and RT-PCR was conducted on Roche LightCycler 480 II (Roche, Basel, Switzerland) with the related primers containing SYBR Premix Ex Taq (TaKaRa, Dalian, China). RT-PCR was repeated three times. We calculated the relative fold changes in the target gene expression via the formula: 2^−△△CT^. The primer sequences adopted in this study are as follows: TNF-*α* forward, 5′‐CGTCCCTCTCATACACTGGC‐3′ and reverse, 5′‐AATTCTGAGCCCGGAGTTGG‐3′; IL-6 forward, 5′‐CCACCCACAACAGACCAGTA‐3′ and reverse, 5′‐GTCTTGGTCCTTAGCCACTCC‐3′; and GAPDH forward, 5′‐CTCTCTGCTCCTCCCTGTTC‐3′ and reverse, 5′‐TACGGCCAAATCCGTTCACA‐3′.

### 2.7. Statistical Analysis

Results are presented as the mean ± standard deviation (SD). Differences among groups were analysed with one‐way ANOVA. When comparing two groups, the LSD method was adopted; when the assumption of equal variance did not hold true, the Dunnetts' T3 method was applied. Data were analysed using the SPSS version 20.0 software (SPSS Inc., Chicago, IL). *p* < 0.05 was considered statistically significant.

## 3. Results and Discussion

### 3.1. Effects of RES on H9c2 Cell Viability

**Figure 1:**
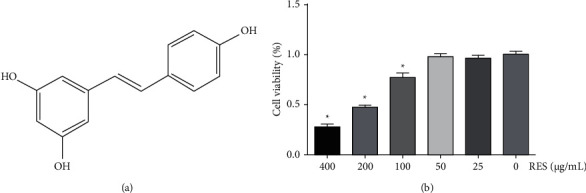
Effects of RES on HG-stimulated H9c2 cell viability. Cells were subjected to treatment with different concentrations (0–400 *μ*g/mL) of RES for 24 h, then assayed with a CCK-8 kit. (a) Chemical structure of RES; (b) cell viability. The data are shown as the mean ± SD of the three repeated trials; ^*∗*^*p* < 0.05 vs. 0 *μ*g/mL (RES) group.

The results of the CCK-8 assay presented that in comparison with the negative control (RES 0 *μ*g/mL), RES did not exhibit significant cytotoxic or stimulatory effects on H9c2 cells up to a concentration of 50 *μ*g/mL after culture for 24 h (*p* > 0.05) (Figure 1(b)). Therefore, we selected 40 *μ*g/mL as the highest concentration for further study.

### 3.2. RES Ameliorates HG-Induced Cardiac Hypertrophy

**Figure 2:**
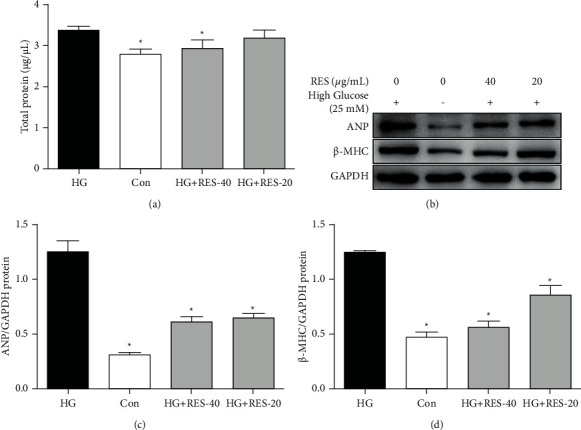
RES's effects on HG-induced hypertrophy in H9c2 cells. Cells were subjected to treatment with control medium or HG containing 20 and 40 *μ*g/mL RES for 24 h. The BCA method and WB analysis of the content of total protein content in H9c2 cells for ANP and *β* -MHC were performed. (a) Content of total protein content in H9c2 cells; (b) representative WB images of ANP, *β* -MHC, and GAPDH; (c) ANP'S relative protein expression; (d) *β* -MHC's relative protein expression. The levels of protein were relatively determined by densitometry and normalised to the level of GAPDH. The data are presented as the mean ± SD of the three repeated trials; ^*∗*^*p* < 0.05vs. HG group.

To investigate whether HG induces hypertrophy in H9c2 cells, the content of total protein in HG-treated H9c2 cells was quantified. HG treatment obviously raised this content in H9c2 cells (Figure 2(a)). In addition, we also determined the expressions of cardiac hypertrophy markers, such as atrial natriuretic peptide (ANP) (Figures 2(b) and 2(c)) and beta myosin heavy chain (*β*-MHC) (Figures 2(b) and 2(d)). Both of them were significantly reduced following the RES treatment in the H9c2 cells.

### 3.3. RES Attenuates HG‐Induced Myocardial Apoptosis

**Figure 3:**
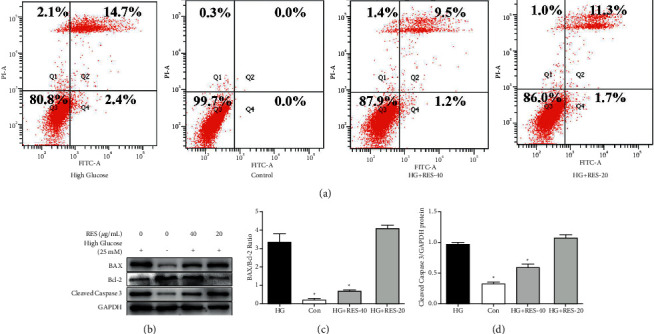
Effects of RES on cell apoptosis in HG-treated H9c2 cells. H9c2 cells were subjected to treatment with control medium or indicated concentrations of RES (20 and 40 *μ*g/mL) for 24 h. Flow cytometry and WB analysis of cell apoptosis markers, Bax, Bcl‐2, cleaved caspase‐3, and GAPDH were conducted. (a) Cell apoptosis of H9c2 cells; (b) representative WB images of these markers; (c) ratio of Bax/Bcl‐2; (d) protein expression of cleaved caspase‐3. The levels of protein were determined by densitometry and normalised to the levels of GAPDH. The data are presented as the mean ± SD of the three repeated experiments; ^*∗*^*p* < 0.05vs. HG group.

After HG incubation for 24 h (25 mM), significant induced cell apoptosis was observed in H9c2 cells, in comparison with that in the control group (Figure 3(a)). However, RES treatment markedly inhibited HG‐induced cell apoptosis (Figure 3(a)). Additionally, RES treatment obviously decreased HG‐induced Bax/Bcl‐2 ratio and cleaved caspase‐3 (Figures 3(b)–3(d)). Collectively, these findings demonstrated that RES prevents HG-induced mitochondria‐mediated cardiac apoptosis.

### 3.4. RES Decreased HG‐Stimulated Inflammatory Cytokine Production and Expression of RAGE and NF-*κ*B Pathways

**Figure 4:**
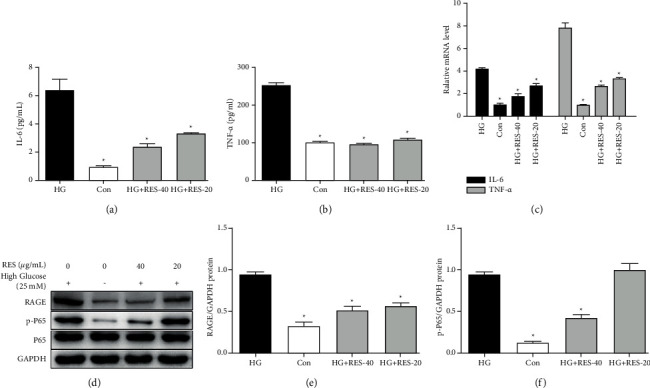
RES's effects of on HG-stimulated activation of the RAGE/NF-*κ*B and inflammatory cytokines production. H9c2 cells were subjected to treatment with control medium or suggested concentrations of RES (20 and 40 *μ*g/mL) for 24 h. ELISA, RT-PCR, and WB analysis of IL-6, TNF-*α,* RAGE, p-NF-*κ*B P65, P65, and GAPDH levels were performed. (a) IL-6's secretion level of IL-6 in supernatant; (b) TNF-*α*'s secretion level in supernatant; (c) IL-6 and TNF-*α*'s mRNA levels in H9c2 cells; (d) representative WB images of RAGE, p-P65, P65, and GAPDH; (e) RAGE's protein expression; (f) p-P65's protein expression. The levels of protein were determined by densitometry and normalised to the level of GAPDH. We normalised the relative mRNA levels to that of GAPDH. The data are presented as the mean ± SD of the three repeated trials; ^*∗*^*p* < 0.05vs. HG group.

The levels of protein and mRNA of TNF-*α* and IL-6 were determined with ELISA kit and RT-PCR. The IL-6 and TNF-*α*'s protein levels in supernatant markedly augmented after the HG stimulation, and this increase was markedly suppressed by RES treatment (Figures 4(a) and 4(b)). Similarly, HG inducement obviously increased IL-6 and TNF-*α*'s mRNA expression in H9c2 cells, while RES treatment (20 and 40 *μ*g/mL) weakened this increase. In order to determine if the pathways of RAGE and NF-*κ*B played an important part in HG stimulation and RES treatment, the levels of protein of RAGE, p-NF-*κ*B p65, and NF-*κ*B p65 were determined using WB. As shown in Figures 4(d)–4(f), HG upregulated the expression of RAGE, and this upregulation was inhibited by RES treatment (20 and 40 *μ*g/mL). Similarly, p-NF-*κ*B p65's expression rose in HG-induced group, and further treatment with RES (20 and 40 *μ*g/mL) suppressed that of p-NF-*κ*B p65 (Figure 4(f)). The expression of NF-*κ*B p65 in H9c2 cells treated with the treatment of HG and/or RES did not change significantly.

### 3.5. RES Alleviated Cardiac Fibrosis Induced by HG In Vitro

**Figure 5:**
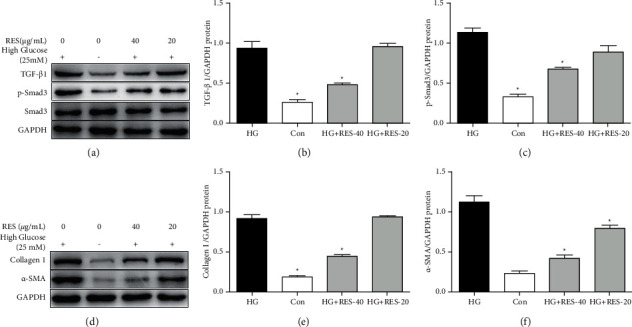
Effects of RES on activation of the TGF‐*β*1/Smad3 pathway and ECM components induced by HG. H9c2 cells were subjected to treatment with control medium or suggested RES concentrations (20 and 40 *μ*g/mL) for 24 h. WB analysis of TGF‐*β*1, p-Smad3, Smad3, collagen 1, *α*-SMA, and GAPDH levels was performed. (a, d) Representative WB images of TGF‐*β*1, p-Smad3, Smad3, collagen 1, *α*-SMA, and GAPDH levels; (b) protein expression of TGF‐*β*1, (c) p-Smad3, (e) collagen 1, and (f) *α*-SMA. The levels of protein were determined by densitometry and normalised to the levels of GAPDH. We normalised the relative mRNA levels to the levels of GAPDH. The data are presented as the mean ± SD of the three repeated trials; ^*∗*^*p* < 0.05vs HG group.

To verify the antifibrotic abilities of RES, we determined the variations in the fibrotic proteins' expression in HG‐treated H9c2 cells. As illustrated in Figure 4, the profibrotic factor TGF‐*β*1, the cardiac fibrosis‐associated protein *α*‐SMA, and the ECM content collagen I were significantly upregulated in HG‐stimulated cells in comparison with those in the control group, as quantified by WB (Figures 5(a), 5(b), and 5(d)–5(f)). However, the expressions of proteins of TGF‐*β*1, *α*‐SMA and collagen I levels were downregulated following the RES treatment (Figures 5(a), 5(b), and 5(d)–5(f)). Additionally, HG stimulation significantly increased p‐Smad3 expression, which was consequently inhibited by RES administration (Figure 5(c)). The change in Smad3 expression was insignificant in H9c2 cells with the treatment of HG and/or RES.

### 3.6. RES Attenuated the HG‐Induced Activation of the NF-*κ*B and TGF‐*β*1/Smad3 Pathways in a RAGE-Dependent Manner

**Figure 6:**
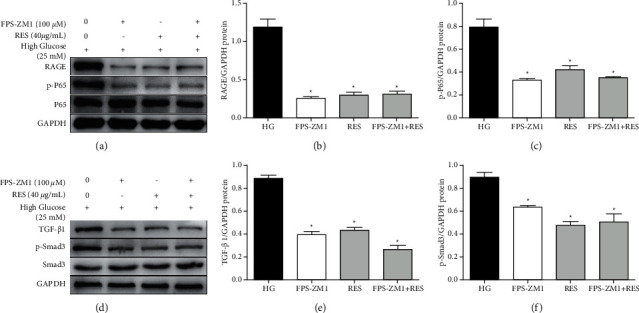
RES suppressed the HG‐induced activation of pathways of TGF‐*β*1/Smad3 and NF-*κ*B dependent on RAGE *in vitro*. H9c2 cells were treated with control medium or indicated concentration of RES (40 *μ*g/mL) or FPS-ZM1 (100 *μ*M) for 24 h. WB analysis of RAGE, p-NF-*κ*B P65, P65, TGF‐*β*1, p-Smad3, Smad3, and GAPDH levels was performed. (a, d) Representative WB images of RAGE, p-NF-*κ*B P65, P65, TGF‐*β*1, p-Smad3, Smad3, and GAPDH levels; (b) protein expression of RAGE, (c) p-NF-*κ*B P65, (e) TGF‐*β*1, and (f) p-Smad3. The levels of protein were determined by densitometry and normalised to the levels of GAPDH. The data are presented as the means ± SD of the three repeated experiments; ^*∗*^*p* < 0.05 vs, HG group.

To validate the influence of RAGE in RES-induced inhibition of NF-*κ*B and TGF‐*β*1/Smad3 pathways, H9c2 cells were treated with the RAGE inhibitor (Figure 6). Using WB, we found that H9c2 cells treated with HG induced a remarkable increase in levels of RAGE. However, this increase could be suppressed by RES treatment. Then, we found that FPS-ZM1 (a RAGE inhibitor) could downregulate NF-*κ*B and TGF‐*β*1/Smad3 pathways (Figures 6(a)–6(f)), while cotreatment with RES and FPS-ZM1 showed inhibitory effects similar to RES treatment alone on these pathways (Figures 6(a)–6(f)). In summary, these data showed that RES may attenuate HG‐induced cardiotoxicity via NF-*κ*B and TGF‐*β*1/Smad3 pathways in a RAGE-dependent manner.

## 4. Discussion

Diabetic cardiomyopathy is a common diabetic complication caused by long-term hyperglycaemia in diabetic patients. The early stage is mainly characterised by oxidative damage and myocardial hypertrophy, which eventually leads to heart failure, arrhythmia, myocardial infarction, and an increased incidence of fragmentation [13, 14]. The pathogenesis of diabetic cardiomyopathy is complicated; no specific drug has been indicated for the treatment at present. RES is a class of nonflavonoid polyphenol compounds, which has the functions of scavenging free radicals, anti-infection, and reducing lipid synthesis [15]. It has been reported that RES can inhibit cardiomyocyte oxidative damage and autophagy induced by high glucose and then reduce cardiomyocyte apoptosis. Other studies have shown that cell hypertrophy caused by high glucose damage is closely related to the outbreak of oxygen free radicals. Additionally, RES can inhibit early kidney damage in diabetic rats and reduce kidney hypertrophy by activating the AMPK pathway [16, 17]. Herein, we found that RES significantly ameliorated HG‐induced cardiotoxicity through suppressing inflammatory response and ECM accumulation via inhibition of RAGE-dependent NF-*κ*B and TGF‐*β*1/Smad3 signalling pathways. The cardioprotective roles of RES were confirmed by several observations. First, RES ameliorated HG-induced cardiac hypertrophy. Second, we demonstrated that RES alleviated HG‐induced mitochondria‐dependent apoptosis. Third, RES treatment significantly decreased the inflammatory cytokine levels and inhibited HG‐induced inflammatory response. Additionally, RES treatment greatly decreased the accumulation of ECM and suppressed HG‐induced cardiac fibrosis. In terms of mechanisms, we identified that RES remarkably reduced HG‐induced NF-*κ*B and TGF‐*β*1/Smad3 signalling through downregulation of RAGE. Thus, these pathways' suppression might be crucial for the cardioprotective roles of RES on HG‐induced cardiotoxicity.

It is widely acknowledged that diabetes increases the risk of developing cardiac vascular disease and hyperglycaemia is the pathogenesis of cardiac vascular disease [18]. Recently, increasing studies have proved that inflammation plays a pivotal role in developing diabetic cardiomyopathy [19, 20]. Inflammation is characterised by an increase in the inflammatory cell number and lifted expression levels of chemokines and inflammatory factors [21]. In an *in vivo* study, BAY 11–7082 (a selective NF-*κ*B inhibitor) protects rats from diabetic nephropathy through decreasing the expression of inflammatory factors such as TNF-*α* and IL-6 and suppressing the oxidative damage regulated by hyperglycaemia [22]. In this study, we indicated that RES suppressed the expression levels of HG-stimulated inflammatory cytokines including TNF-*α* and IL-6 in H9c2 cells, suggesting that RES protected against HG-stimulated inflammation.

Cardiac fibrosis is common among various cardiac pathophysiological states and has been thought to be closely associated with stiffness and dysfunction of the heart in HG-stimulated cardiotoxicity [23]. Herein, we demonstrated that treatment with RES significantly suppressed the TGF‐*β*1/Smad3 pathway, reduced ECM, and alleviated fibrosis in HG‐stimulated cells. This pathway, as the most powerful and ubiquitous profibrogenic cytokines, plays an essential role in the development of fibrosis [24, 25]. It was also known that HG promoted the progression of fibrosis by regulating collagen I, collagen III as well as *α*‐SMA [26]. Consistently, our results implied that the protein expressions of *α*‐SMA and collagen I were greatly overexpressed in HG‐stimulated H9c2, suggesting cardiac fibroblast activation and excessive ECM deposition. Following RES treatment, the levels of ECM proteins (*α*‐SMA and collagen I) were dramatically downregulated.

Apoptosis also contributes to the development of cardiac dysfunction [27]. Therefore, targeting myocardial apoptosis may be an effective target for patients with diabetic cardiomyopathy. H9c2 cardiomyocytes are a cell line derived from an embryonic rat heart whose biological functions are similar to those of primary cardiomyocytes [28]. Nevertheless, it is unclear whether RES can protect the H9c2 cells from HG-stimulated damage. Herein, we discovered the HG-induced apoptosis of H9c2 cells was significantly alleviated by RES treatment.

Based on the literature review, both ECM accumulation and inflammation are thought as the main aetiologies underlying the pathogenesis and progression of diabetic cardiomyopathy [29]. Increasing research has demonstrated that RAGE's mechanisms and related ligand families show vital effects in diabetes pathogenesis and its *in vivo* and *in vitro* complications. It is reported that RAGE has established a link between inflammation and fibrosis in diabetic nephropathy [12, 30]. Additionally, increasing studies have recently found that the receptor for RAGE shows a vital effect in mediating myocardial ischemia-reperfusion injury. RAGE, an immunoglobulin superfamily member, is one of the molecules on the cell surface; it can bind to a variety of ligands and affect intracellular signal transduction to stimulate the release of cytokines and then exert its biological effects. Reperfusion injury and inflammation are closely related. Studies have confirmed that HMGB1 interacts with RAGE to promote NF-*κ*B activation and amplify the inflammatory response in myocardial ischemia-reperfusion injury. NF-*κ*B belongs to a transcription factor family, which is closely associated with different physiological processes, particularly in inflammatory and immune responses [31]. In ischemia-reperfusion injury, upregulation of RAGE leads to the death of cardiomyocytes, while knockdown of RAGE or si-RAGE downregulates the expression of RAGE, which can reduce oxidative stress, inflammation, and apoptosis and protect cardiomyocytes and tissues. These shreds of evidence indicate that inhibition of these pathways may show a contributing role in preventing inflammatory diseases. *In vivo*, RAGE ligands were significantly upregulated in a diabetic cardiomyopathy apoE−/− mouse model in comparison with controls (nondiabetic apoE−/−) [32]. Additionally, diabetic RAGE−/−/apoE−/− mice (double knockout) have been demonstrated to reduce leukocyte recruitment and decrease indicators of proinflammatory and oxidative stress [33]. Moreover, in clinical studies, it has also been proved that improved expression of the AGE-RAGE axis was dramatically and powerfully associated with cardiovascular dysfunction and vascular endothelial damage [34]. Therefore, there is an urgent need to develop therapeutic agents with molecular mechanisms involved in RAGE and related downstream signalling specific for the developing diabetic cardiomyopathy. Collectively, our findings from *in vitro* investigations elucidated a novel mechanism underlying the effect of RES in diabetic cardiac vascular disease; RES treatment obviously decreased RAGE expression in H9c2 cells. Furthermore, using FPS-ZM1 (a selective inhibitor of RAGE) which reversed the HG-induced inflammatory responses and ECM accumulation, we confirmed that RES showed protective effects on the heart through RAGE-dependent pathways. However, this study is restricted to *in vitro* studies, and further studies should be conducted to support our conclusion.

## 5. Conclusions

**Figure 7:**
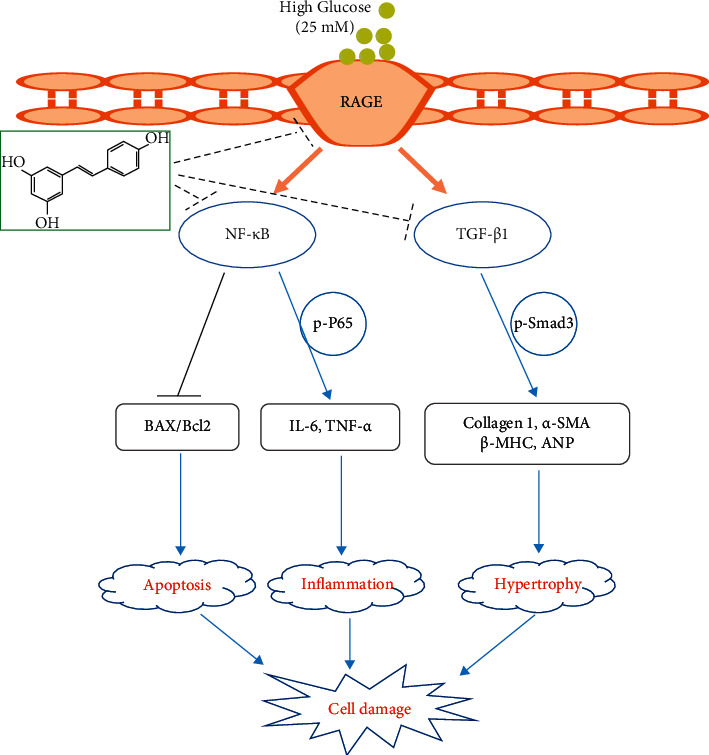
Schematic drawing of the cardioprotective effects of resveratrol on HG‐stimulated H9c2 cardiomyocyte damage.

Our data showed that RES treatment protects against hypertrophy, inflammatory response, fibrosis, and apoptosis induced by HG, eventually enhancing cardiac function in H9c2 cells treated with HG. The RES's cardioprotective effect arises from its ability to inhibit NF-*κ*B and TGF‐*β*1/Smad3 pathways in a RAGE-dependent manner (Figure 7). Our results demonstrated that RES might be a potential novel therapeutic agent, which can protect against HG-induced cardiotoxicity. However, additional evidence from future studies involving animals is required to confirm the role of RES in cardiovascular diseases.

## Data Availability

The data used to support the findings of this study are available from the corresponding author upon request.

## Abbreviations

RES: Resveratrol
RAGE: Receptor for advanced glycation endproducts
TGF‐*β*1: Transforming growth factor beta 1
HG: High glucose
IL: Interleukin
TNF-*α*: Tumour necrosis factor alpha
NF-*κ*B: Nuclear factor kappa B
ANP: Hypertrophic biomarkers like atrial natriuretic peptide
*β*-MHC: Beta-myosin heavy chain.
